# Platformized visibility and diverse discourses of depression discussions on Weibo

**DOI:** 10.3389/fpsyg.2026.1829252

**Published:** 2026-08-06

**Authors:** Na Liu, Shiyi Zhang

**Affiliations:** 1 College of Humanities and Law, Zhejiang A&F University, Hangzhou, China; 2 School of Journalism & Communication, Chongqing University, Chongqing, China

**Keywords:** depression, discursive interaction, mental health, social media, visibility

## Abstract

**Introduction:**

This study examines discursive dynamics of depression on Weibo and the digital visibility of depressive experiences. Building on critical discourse analysis (CDA), we develop a platformized discourse analysis approach that combines purposive stratified sampling across Weibo’s visibility layers with CDA to examine how platform mechanisms shape the circulation and regulation of depression discourses.

**Methods:**

The dataset comprises two policy documents, 300 posts, and 900 comments and reposts collected between January and August 2023 from four categories of visibility contexts, including state-media accounts, individual users, celebrity-related discussions, and depression-focused super-topic communities.

**Results:**

The findings reveal three main discourses: official timely detection and intervention, individual freely venting fragile sentiments, and community-connected resonance and rationalization.

**Discussion:**

This paper introduces the concept of “manifesting depressive agency” to describe the ongoing process through which individuals and communities claim, negotiate, and sustain the visibility and legitimacy of depressive experiences within platformized public discourse. By conceptualizing agency as continuous negotiation, this study advances understanding of how mental-health discourses emerge within the interplay of platform power, institutional governance, and user practices.

## Introduction

The digital technologies have profoundly reshaped the landscape of health communication, with social media emerging as a critical approach for individuals to discuss sensitive topics, particularly mental health (Li and Xu, 2025a). Sharing experiences of depression on social media can reshape public discourse on mental illness (Wang and Liu, 2016; Pan et al., 2018; Zhang et al., 2023). Existing studies on depression discussions on social media have focused on various aspects, such as the sense-making of depression (Kotliar, 2016; Xu and Li, 2023), depression sufferers’ identity reconstruction (Li and Xu, 2025b), coping (Li and Xu, 2023), self-stigma (Gu et al., 2026), the community construction among people with depression (Li and Xu, 2025a), the impact of algorithms on connectedness for individuals with depression (Wang et al., 2023), and the influence of celebrity depression on public discussions (Francis, 2018; Hoffner, 2020; Leung, 2019; Park and Hoffner, 2020). However, three issues remain underexplored. First, studies of user communities and studies of institutional framing have largely proceeded in isolation, leaving the interplay between state governance and user agency in shaping online mental-health discourse poorly understood. Second, although visibility is central to how social media operates, little attention has been paid to how a platform’s visibility architecture determines which depression discourses become perceptible, and how censorship renders others invisible. Third, the prevailing reliance on keyword-based sampling tends to overlook censored or low-visibility voices, precisely those most exposed to state intervention.

In China, the prevalence of mental health issues among adults is reported to be around 17.5%, where the proportion for depression is about 6.1% (Que et al., 2019). Depression disorder has long been stigmatized in Chinese culture (Haraguchi et al., 2009). As the most widely used public discussion sphere in China, Sina Weibo is an ideal site for examining the discourse and visibility dynamics of depression on social media (Zhang et al., 2021), as Weibo hosts a significant amount of discussions on depression (Pan et al., 2018).

This study addresses these gaps by treating platform visibility as the organizing lens, bringing governmental discourse on depression in China (e.g., Zhang et al., 2021) into the same analytical frame as the individual and community voices whose visibility is shaped by China’s internet censorship (Yang, 2016; Yang et al., 2007). Understanding the discourse and visibility dynamics of depression on social media is essential for fostering supportive dialogues around mental health. Focusing on China’s social media landscape contributes to the existing research in debating the state’s governance and social work of digital health communication. This study focuses on two research questions:

*RQ1: How do the visibility mechanisms of Weibo structure the competing official and vernacular discourses of depression?*

*RQ2: How do Weibo users navigate and negotiate the digital visibility of their depressive experiences and enact agency against state and platform censorship?*

To answer these questions, a diverse dataset, including posts from government accounts, individual narratives, and various discussions about depression, was built. We propose platformized discourse analysis as a sampling framework to examine how social media platform visibility structures shape discursive formations. Our findings offer novel insights into the mechanisms through which state and citizens co-construct the public discourses on depression. This paper introduces the concept of “manifesting depressive agency,” which signifies the ongoing process through which individuals and communities claim, negotiate, and sustain the visibility and legitimacy of depressive experiences within platformized public discourse. The concept highlights how users actively express vulnerability, participate in social meaning-making, and construct identities through strategic engagement with platform affordances while navigating institutional visibility regimes.

## Literature review

### Evolving discourses of depression

In the Chinese context, mental illness has long been highly stigmatized (Gu et al., 2026; Haraguchi et al., 2009; Li and Xu, 2025b). Traditional beliefs attribute mental health issues to supernatural causes, such as punishment from ancestors or spirits (Haraguchi et al., 2009). Mental illness is seen as a moral failing or result of bad karma, which fosters societal negative attitudes to those with mental problems. As a result, individuals suffering from mental diseases often face social distancing and rejection from their communities, friends, and even family members (Haraguchi et al., 2009). Collectivist social norms, influenced by traditional Chinese culture, place a strong emphasis on maintaining “face” (social reputation). Mental issues may negatively affect a family’s reputation, as disclosing conditions such as depression is often perceived as shameful and humiliating, particularly in front of familiar individuals (Haraguchi et al., 2009). Furthermore, political ideologies of the past have also shaped the stigmatization of mental illness. “In the socialist past, many cases of mental illness were attributed to incorrect political thought and could only be cured through political education” (Yan, 2010, p.506).

Confucian values and Chinese party-state’s emphasis on social harmony and positive mainstream narratives lead to the suppression of “negative” content (Yang, 2016; Yang et al., 2007). Although the Chinese government has taken steps to promote mental health awareness through psychological interventions and regulatory measures, these efforts primarily frame mental health as an individual rather than a structural issue (Pan et al., 2018). Previous research implies that both the state-owned media and market-owned media primarily attribute depression to individual-level factors, emphasizing personal responsibility in managing and overcoming mental health conditions (Zhang et al., 2021). This individualization of mental health problems can reinforce public perceptions of depression as a personal weakness rather than a treatable health condition, further perpetuating stigma (Zhang et al., 2021). Reading through Foucault’s biopolitics and governmentality (Foucault, 1978, 1991), this individualizing frame functions less as a cultural residue than as a technique of government, enlisting citizens in the management of their own conduct while deflecting attention from structural conditions (Rose, 1999). In the Chinese context, this responsibilizing logic is folded into party-state discourse of social harmony and positive mainstream narratives, so that expressions of distress that expose disharmony become politically sensitive (Yang, 2016; Yang et al., 2007).

Digital platforms serve as a crucial venue for the transformative discourses of mental illness, showing the possibility of destigmatizing mental illness. Due to the anonymity of the platforms, ordinary people are generally more willing to share their experiences of suffering from mental illness, including causes and symptoms of the issues, as well as their emotional states and daily struggles (Kotliar, 2016; Pan et al., 2018; Wang et al., 2023; Xu and Li, 2023). They also frequently discuss coping strategies, ranging from personal efforts like self-help and lifestyle changes to seeking medical or psychological assistance (Li and Xu, 2023; Pan et al., 2018).

Digital platforms allow ordinary people with depression to discuss their experiences while maintaining social distance, fostering a sense of understanding emotions. This “imagined intimacy” (Hendry, 2020, p.1) is amplified by algorithms that create “algorithmically woven communities” (Wang et al., 2023, p.1), connecting users with similar experiences. On Chinese social media, young people exchange narratives of depression, finding peer support and knowledge within these digital communities (Li and Xu, 2025a; Wang and Liu, 2016). The disclosure of personal depressive feelings, combined with collective encouragement for positive mutual support, fosters the creation of a sustainable community (Li and Xu, 2025a).

Celebrity involvement in mental health advocacy can significantly enhance visibility and reduce stigma by normalizing discussions surrounding mental health issues (Leung, 2019). The death of a celebrity due to mental health struggles or a celebrity’s public disclosure of their own mental health challenges often catalyze widespread public discourse (Zhang et al., 2024). These events can lead both fans and the general public to share their own mental health experiences (Hoffner, 2020; Park and Hoffner, 2020). Such occurrences underscore the influential role that celebrities can play in shaping public conversations about mental health, fostering greater openness, and helping to reduce stigma (Francis, 2018; Hoffner, 2020; Leung, 2019; Park and Hoffner, 2020).

### Digital visibility on social media

Visibility is generally understood as the potential for perceptibility of subjects and their social behaviors (Thompson, 2005). Prior to the emergence of mass media such as newspapers and broadcast, visibility was confined to the limits of physical sights, meaning that our perception was bound by our immediate spatial–temporal context (Thompson, 2005). With the advent of technology, visibility started to extend beyond physical limits while being mediated by gatekeepers like journalists and editors in mass media agencies (Li et al., 2024; Thompson, 2005).

Digital visibility concerns the visibility of data, people, or activities that are visible to others in digital spaces (Zhang et al., 2023). The popularization of digital technologies has reshaped the forms of interaction and power dynamics of visibility (Thompson, 2005). These dynamics unfold within the platformization of public communication, the process through which platforms’ data-driven, algorithmic, and commercial infrastructures reorganize how information circulates and is governed (van Dijck et al., 2018; Poell et al., 2019). Within this infrastructure visibility is not a neutral property but an allocated resource whose distribution is itself a modality of power (Brighenti, 2007), one that operates as much through the threat of invisibility as through exposure, since content that is not amplified is effectively silenced (Bucher, 2012).

On the one hand, digital technologies have enhanced those in power. On digital platforms, the flow of information is deeply influenced by algorithms designed based on human subjectivity and values (Wang et al., 2023; Yin and Xie, 2024). This has made it convenient for the digital platforms and those in power to control the information flow by shaping algorithm design. For example, Weibo is required by the Chinese government to reserve a space above all ranks on its Hot Search List, which is often used to promote positive content by the government (Cui and Kertész, 2023). Such arrangements exemplify platform governance, the interplay of state regulation, platform rules, and automated moderation that shapes what remains sayable online (Gillespie, 2018; Gorwa, 2019). In China, this governance operates through mandated self-censorship, so that the platform functions at once as a commercial actor and as an extension of state visibility management (Yang, 2016).

On the other hand, digital technologies have also created opportunities for ordinary people and challenges for those in power (Li et al., 2024). Social media empower users to generate and disseminate their own content to a broad audience rapidly (Poell et al., 2014), and to co-define digital visibility through interactions with the platforms (Zhang et al., 2023). Moreover, users can foster “networked acknowledgement” by forming online communities, contributing to the legitimization and amplification of visibility of their content (Li et al., 2024, p.5). This provides more possibilities for ordinary people to negotiate their digital visibility.

The study by McCosker and Gerrard (2021) indicates that discussions under depression-related hashtags on Instagram often convey a “depressed aesthetic” (p. 1900) or “sad aesthetics” (p. 1908). These hashtags make depression more visible, allowing individuals to negotiate their identity, create new conversations, and generate resonance. They also foster a “complex collectivism” (p. 1910), where individuals collectively navigate and express their experiences. In societies like China where regulations are tightly imposed on online information, the netizens employ some specific methods to bypass the regulations to enhance their visibility such as re-coding or using homophones of regulated censored keywords (Yang, 2016). Thus, the empowerment of ordinary people by social media has shifted the power dynamics related to visibility and control (Poell et al., 2014; Thompson, 2005).

Social media platforms provide a connected infrastructure that enables ordinary users to communicate, engage in public event discussions, and participate in the shaping of discourse (Yin and Xie, 2024). The visibility of news, the resonance of activism, and the networked legitimacy of groups on social media contribute to the discursive opportunities of social media activism (Li et al., 2024). The technological features of platformization, particularly visibility mechanisms, significantly influence user participation (Yin and Xie, 2024). Conversely, users can also influence the visibility of their posts by strategically manipulating engagement metrics such as likes, shares, and comments (Zhang et al., 2023; Yin and Xie, 2024).

**Figure 1:**
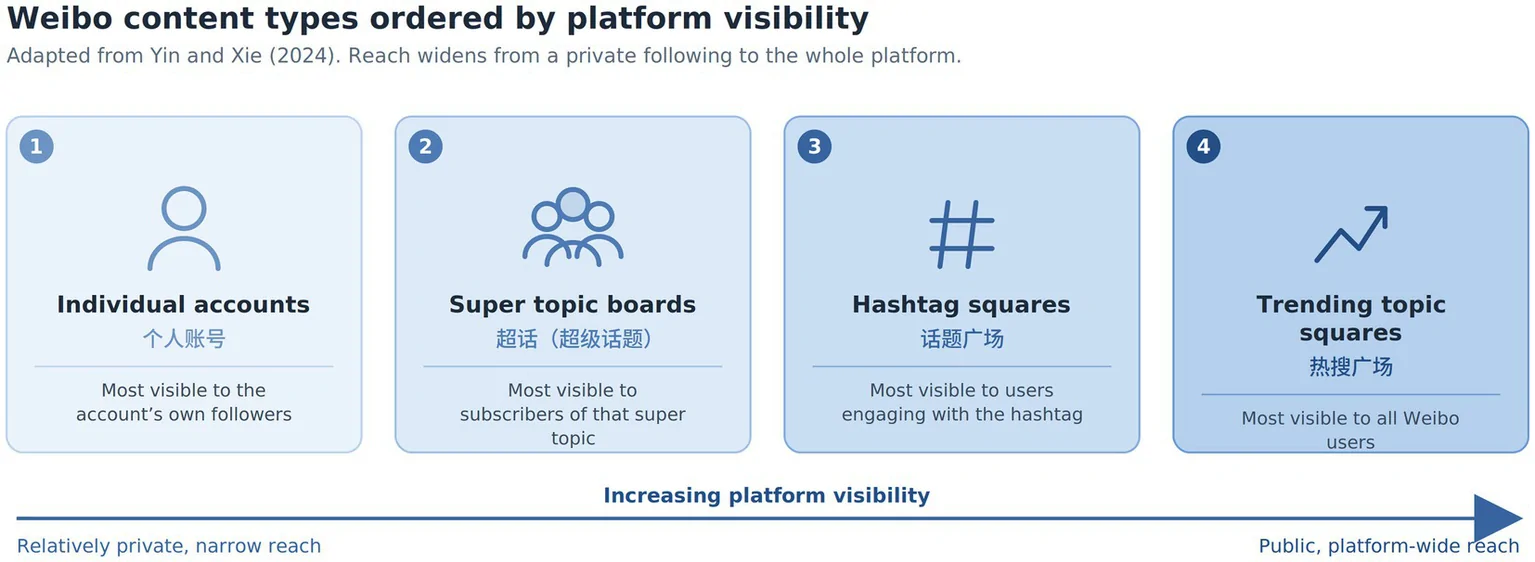
Four types of Weibo content, ordered by increasing platform visibility.

The technical characteristics of Weibo, its platform’s self-censorship, government interventions, and user cultures mutually influence each other, forming a platform for different discourses (Poell et al., 2014). Yin and Xie (2024) categorize the visibility of Weibo content into four types (Figure 1), based on the platformized visibility mechanisms: individual accounts, which are most visible to their followers; super topic boards, which are most visible to super topic followers; hashtag squares, which are most visible to relevant participants; and trending topic squares, which are most visible to all Weibo users.

Platformization structures visibility, visibility conditions which discourses become sayable, and discourse is itself a site where power is exercised and contested (Fairclough, 2010; van Dijk, 2006). Platform users’ agency is a capacity enacted through acts that intervene in the distribution of platform visibility. This resonates with work on digital citizenship, in which citizens come into being through claims made in and through digital media rather than through a status granted in advance (Isin and Ruppert, 2015). Building on this, we develop the concept of manifesting depressive agency. We define it as the ongoing process through which individuals and communities claim, negotiate, and sustain the visibility and legitimacy of depressive experiences within platformized public discourse. It captures the need, the right, and the autonomy to make depressive experiences visible, to participate in public discourse, and to construct their identities.

Users advance this claim in negotiation with the platform’s visibility rules and institutional regulation of negative emotional expressions. The concept treats platformization as the infrastructure of visibility, visibility as the stake of action, discourse as the medium in which depression is defined, and agency as citizenship enacted rather than granted. It is this negotiated and visibility-oriented character of depressive agency that our analysis sets out to trace.

## Materials and methods

### Data collection

Driven by the authors’ research interest in online depression discourse since 2019 and informed by preliminary observations, this study was ignited by two pivotal events in July 2023: the case of an individual account (pseudonymously referred to as Feiyang) that reported police intervention after sharing personal depressive experiences, and the death of Meiming (pseudonym), a celebrity whose struggle with depression dominated the first trending topic on Weibo. These two events constituted the initial samples for the present qualitative study. Our research methods were designed based on the visibility mechanism of Weibo platform (Yin and Xie, 2024). We assume that all non-private posts that we can publicly access require visibility, as users could otherwise have set them to be visible only to themselves or their friends (Osatuyi et al., 2018). According to Yin and Xie (2024), the visibility structure of Weibo is divided into individual accounts, super topic boards, hashtag squares, and trending topic squares (Figure 1). From the user’s page to the trending topic discussion board, the visibility of layers increases from relatively private to public.

**Figure 2:**
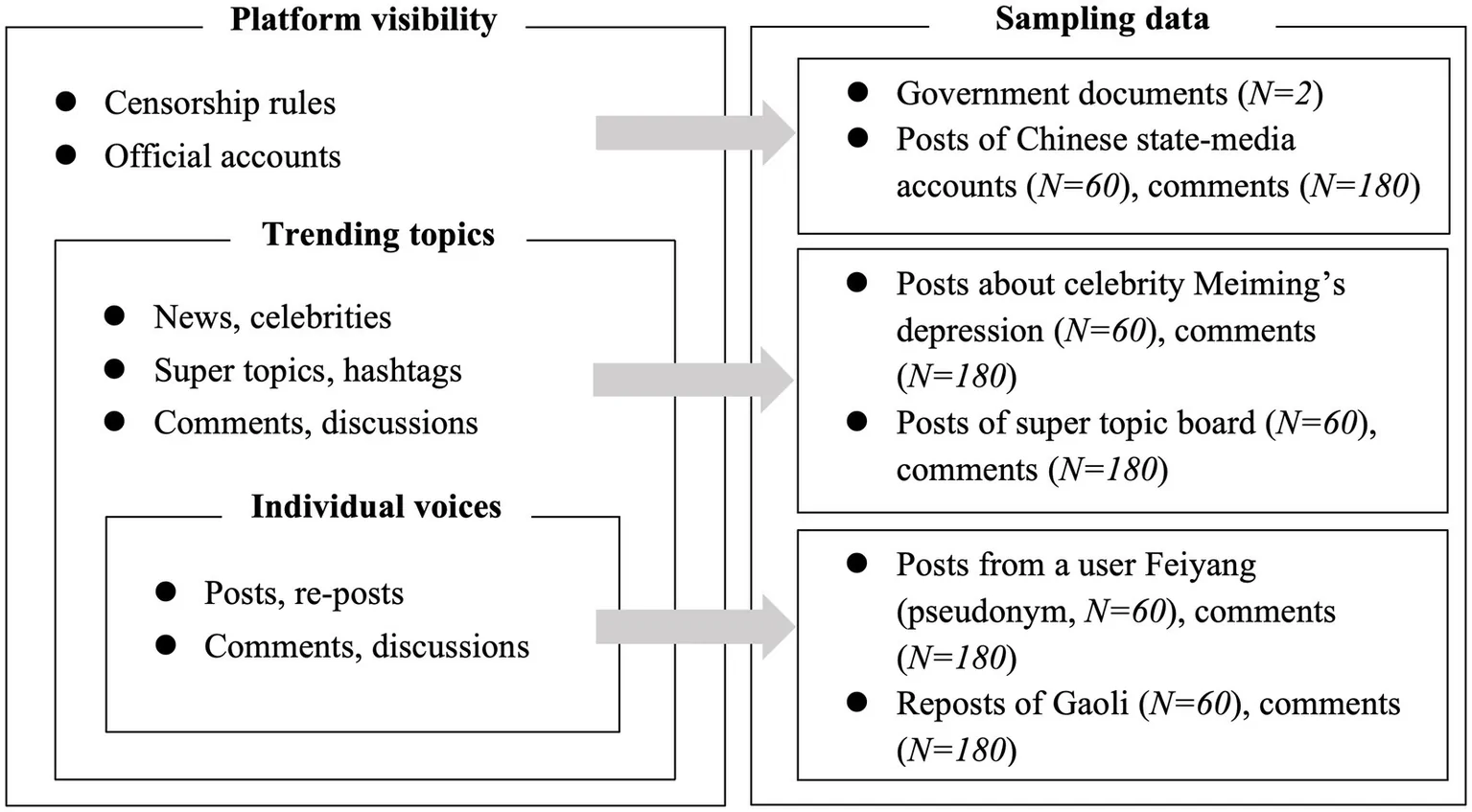
The sampling strategy and data collection based on platform visibility on Weibo.

Our data collection involved scraping publicly available data from Weibo using purposive stratified sampling (see Figure 2). We first summarized five main types based on previous literature on Weibo platform (e.g., Leung, 2019; Li et al., 2024; Park and Hoffner, 2020; Yang et al., 2022; Yin and Xie, 2024). In Yin and Xie’s (2024) classification, hashtag discussions often appear across individual accounts, super topics, and trending topic posts. Therefore, we did not treat hashtag discussions as a separate category for sampling. Within the category of individual accounts, we selected one anonymous user as well as a news figure known for discussing depression. Additionally, we considered the substantial influence of government and platform moderation practices on the visibility of depression-related discussions (Yang et al., 2022).

Chinese social media operates under significant political influence, and Weibo’s visibility mechanisms determine which content remains visible and which is removed as “sensitive” (Yang, 2016). This implicit yet powerful platform rule shapes the contours of public discourse. To capture this point, we included two representative government-affiliated accounts in our sample. We also incorporated two official Chinese government documents related to mental health to help us understand the collected data: Chapter 5, Section 3, ‘Promoting Mental Health’ under ‘Fostering Autonomous and Self-Disciplined Health Behaviors’ in the *Healthy China 2030 Planning Outline* (released in 2016), and the *Guidelines for Depression Prevention and Treatment Services* (2020). In the following, these two documents will be referred to as *Healthy China 2030* and *Guidelines for Depression (2020)*.

The stratified sampling strategy, based on the Weibo platform’s visibility assemblage, allowed us to collect a more diverse and nuanced range of discourses. By sampling across different visibility levels, we were able to account for both highly visible and less visible forms of depression-related discussion. Moreover, this approach enriched the variety of discursive voices in our data and enabled cross-checking of discursive codes. In contrast to purely random sampling based on keyword searches, which may overlook invisible or less visible discursive factors, our strategy offers a more nuanced understanding of how various actors shape depression discourse on Weibo.

For each identified category, we randomly collected an equivalent number of posts discussing depression. The final data include 60 posts of two Chinese state-media accounts discussing depression; 60 posts about celebrity Meiming’s depression; 60 posts of super topic board *yiyuzheng chaohua* (Depression Super Topic); 60 reposts of Gaoli (pseudonym, a university student who committed suicide due to depression, an event that garnered widespread attention on Weibo, with the comment section of her posts previously functioned as a tree hole); 60 posts from a user Feiyang (pseudonym).

The sample size of 60 posts per category was determined through an iterative sampling and analytical process. During the initial data collection, Feiyang’s posts yielded approximately 60 valid posts and 180 comments suitable for analysis. Based on this dataset, we conducted a trial discourse analysis of posts related to Meiming’s depression and reposts of Gaoli. The preliminary coding process indicated that most major themes and discursive patterns had emerged by around the 40th post. We therefore selected 60 posts as a sufficiently information-rich sample size and applied the same sampling threshold across all categories.

This approach is consistent with qualitative inquiry, in which sample adequacy is evaluated not through statistical representativeness but through researchers’ theoretical sensitivity to the research topic and the interpretative depth generated through close engagement with data (van Manen and Beck, 2026). The purpose of this sampling strategy is therefore to capture diverse discursive formations and platform-specific practices surrounding depression discourse on Weibo rather than to achieve quantitative generalizability.

The dataset spans the period from January to August 2023. The keywords for searching the data include ‘depression’ (*yiyu*), ‘mental health’ (*xinli jiankang*), ‘mental problems’ (*xinli wenti*), ‘mental illness’ (*xinli jibing*), ‘psychotherapy’ (*xinli zhiliao*). We collected content from 300 main posts and also examined the comments and reposts, adding 900 comments and reposts to the dataset. Except for the explicitly identified account holders, such as the two state-media accounts and Feiyang, all other posts and comments were collected from different user accounts with distinct identifiers. To capture authentic and substantive user comments, main posts were selected based on the presence of evaluative content or self-disclosure related to depression. After meeting these criteria, posts were randomly selected. However, this sampling standard could not be applied to the account Feiyang. In this case, we selected 60 posts and 180 comments with the highest word count within the specified time range. For comments and reposts, the sampling principle was to select the top three most popular comments under each main post.

### Data analysis

We employed critical discourse analysis (CDA) to examine the data. This analytical approach focuses on the meaning-making practices of discursive power, particularly the (re)production of dominant discourses. It not only seeks to extract discourses but also investigates how they are generated, reproduced, and legitimized within social power structures (Fairclough, 2010). CDA focuses on three key dimensions: text, discursive practice, and social practice. The textual dimension examines the linguistic features of the text; the discursive practice dimension explores the processes of discourse (re)production behind the text; and the social practice dimension situates discourse within the interplay of different discourses and social power (Fairclough, 2010). Building on this framework, we adopt the platformized discourse analysis approach, which integrates platform-visibility sampling strategies with CDA.

Guided by the research questions concerning the discourses, visibility, and user agency in depression discussions, we first identified three major visibility mechanisms on Weibo and organized the data according to three key subjects involved in depression discourse production: the state, individuals, and groups. Within each category, we further examined how different actors constructed meanings of depression, negotiated visibility, and employed various strategies of participation and self-positioning.

Our iterative qualitative analysis using NVivo 12 identified three main discourses: official timely detection and intervention; individual freely venting fragile sentiments; community-connected resonance and rationalization. These discourses were derived from six primary codes: harmonious screening, individualism legitimacy, citizenship protest, vulnerability disclosure, empathetic support, and tactical rationalization. Two researchers independently conducted close readings and coding, drawing on existing literature and repeated engagement with the data. After comparison and discussion, we reached consensus on the final three discursive categories and six primary codes.

**Figure 3:**
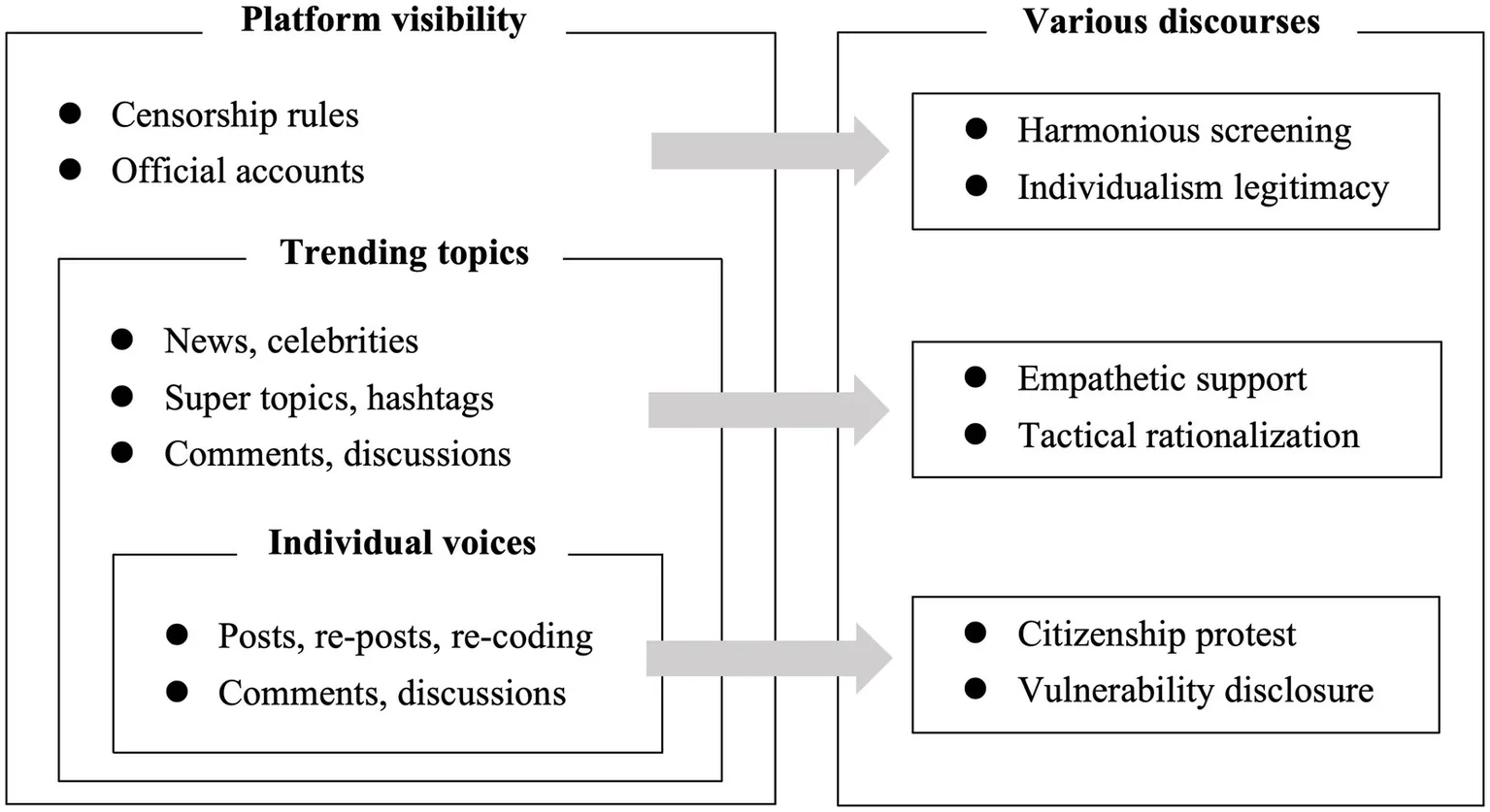
The platformized discourse analysis of depression discussion on Weibo.

In conjunction with the stratified sampling strategy based on platform visibility, we mapped the relationship between the identified discourses and corresponding levels of visibility (see Figure 3). We refer to this integrated approach, combining platform-based stratified sampling and critical discourse analysis, as platformized discourse analysis.

We conducted a second round of coding to further refine the internal variations and discursive patterns within each category. This stage involved a more focused, iterative analysis guided by the emerging findings and the need to elaborate how specific discourses were constructed and negotiated. For example, comments coded under citizenship protest were further categorized into three types of responses, while discussions related to empathetic support were inductively analyzed to identify recurring explanations of the causes of depression. This second-stage coding was not intended to generate new discursive codes but to provide analytical depth. Both rounds of coding were conducted iteratively by the two researchers, who continuously compared interpretations, refined categories, and reached consensus through discussion.

### Ethical report

Considering the sensitive nature of depression and suicidal ideation and to protect users’ privacy, we use pseudonyms or refrain from identifying the authors of quoted materials. Additionally, rather than reproducing the original posts in full, we primarily excerpt relevant segments for analysis. Since the original posts were written in Chinese, all quotations presented in this article are translated into English with minor modifications to reduce traceability while preserving the original meanings and discursive features. These translation and anonymization practices aim to balance analytical transparency with ethical protection of users’ privacy.

This study was reviewed and approved by Media, Communication and Sociology Research Ethics Committee, University of Leicester (Ethics Reference: 31467-sz232-ss/mc:media&communication). Written informed consent was not required, for either participation in the study or for the publication of potentially/indirectly identifying information, in accordance with the local legislation and institutional requirements. The social media data was accessed and analyzed in accordance with the platform’s terms of use and all relevant institutional/national regulations.

## Main findings

### Timely detection and intervention

#### Harmonious screening

The Chinese government’s policy documents on mental health services clearly emphasize “early detection,” “timely intervention,” and “screening and assessment.” In the *Guidelines for Depression (2020)*, the government’s main tasks are listed as “conducting screenings, assessments, and timely interventions.” The guidelines call for a nationwide focus on mental health, encouraging public support for and participation in depression prevention and treatment efforts. The work plan from the National Health Commission highlights the need to target specific groups, such as adolescents, pregnant women, the elderly, and high-stress workers, for screening and follow-up. In Chapter 5, Section 3 of the *Healthy China 2030*, it states,

*(…) Increase the efforts in public mental health education and improve mental health literacy. Strengthen interventions for common mental disorders and psychological and behavioral issues (…) Improve the reporting, registration, treatment, and support management of individuals with severe mental disorders. (…) By 2030, the level of prevention, treatment, and intervention for common mental disorders and psychological and behavioral issues will be significantly improved.*

Governmental accounts echo this discourse of monitoring individuals’ depression, for example:

*#Chengdu comprehensively conducts screening for postpartum depression# (…) The plan is to increase the screening rate to over 90% across the city by 2025.*

This official discourse engages in strategic interdiscursivity. The policy documents appropriate the vernacular language of “care” and “protection” to legitimize intervention.

Weibo user Gaoli posted a final message stating that she intended to commit suicide due to depression. After her death, her last post became a tree hole where many people express their sensitive emotions. Prior to 2022, this post garnered over 2 million comments from 350,000 users, many of which shared their own depressive or suicidal thoughts (Yang et al., 2022). However, from 2022 onwards, the comments under Gaoli’s posts have been heavily censored, being automatically filtered. We tried to leave a comment but received this notification from the system notification: “*Sorry, there is a violation of relevant laws and regulations or the Weibo Community Convention, and the current operation is not possible. [Weibo automatically].”*

It is unclear whether this censorship is solely the platform’s action, a requirement from the government, or a combination of both. However, such censorship discourages free speech, potentially exacerbating the stigma and isolation experienced by individuals with depression. According to media reports, the Institute of Psychology at the Chinese Academy of Sciences uses artificial intelligence to monitor Weibo posts and intervene with users who show suicidal tendencies under Gaoli’s Weibo (Tu, 2019). This practice reflects what can be described as a form of “harmonious screening,” a governmental discursive technique aimed at monitoring and filtering citizens’ negative expressions at scale.

#### Individualism legitimacy

Our data shows that although the official accounts seek to destigmatize the illness, using discourse like “depression is a common brain disease worldwide,” the accounts primarily attribute mental illness to personal issues. They ask individuals to detect early and seek treatment early, and call for people to care for depressive people around them. This discourse of individualism legitimacy places responsibility on individuals rather than on societal services.

Meanwhile, it attributes the primary causes of mental health issues to the individual relationships. As a state-media account posted:

*#What is the root cause of young people’s mental health issues?#* (…) *A host discusses the mental health of young people, stating that the main reasons lie with the older generation, parents, teachers, and the surrounding environment.*

Such narratives exemplify the discourse of individualism legitimacy, through which responsibility for mental health is shifted from structural or institutional provision to individuals and their private networks, rather than being addressed through broader societal or public service mechanisms. This discourse operates through a hierarchical allocation of responsibility: the causes of depression are first located within the family as the primary site of accountability, and subsequently attributed to schools or society at large. In doing so, the state effectively transfers the burden of care onto individuals and families, while collective and institutional obligations toward mental health remain comparatively underemphasized.

While official posts advocate for professional treatment, the information on accessible mental health professionals remains vague for the general public. In our dataset, we found only three instances where specific social services were mentioned, which were limited to providing hotline numbers, such as 12355 Youth Online Service Hotline. The *Guidelines for Depression (2020)* state that “Psychological assistance hotlines should be promoted through various newspapers, TV, radio, and the internet to increase their social influence.” However, it was not until December 2024 that the National Health Commission officially announced that the 12356 hotline would begin operating as the national unified psychological assistance hotline. This news reveals that until this date, the availability of psychological assistance services across the country was inconsistent, shifting the burden of care back to the individuals.

### Freely venting fragile sentiments

#### Citizenship protest

Intense depressive storytelling can ignite emotional struggles, sometimes evolving into sentimental activism. Radical individuals call for digital rights to diverse depictions of depression and the free visibility to exchange ideas, serving as rightful resistance against government over-protective censorship. Our data demonstrates that depressive messages on Weibo are subject to censorship by local governments, thereby corroborating the official discourse of harmonious screening.

Feiyang, a user on Weibo who reported experiencing depression, faced serious repercussions after posting content related to depression and suicidal ideation. Feiyang described being harassed by the local police, who allegedly demanded the deletion of such posts. According to the account, the police frequently contacted family members and subjected them to humiliation. Feiyang maintained that the depression-related content did not violate any laws. Nevertheless, they reported being placed under close surveillance and perceived a lack of freedom of expression in online spaces.

Feiyang’s defense illustrates a form of protective interdiscursivity. When the police framed their distress as political pessimism, Feiyang strategically cited a clinical diagnosis to shield their expression. By borrowing the scientific fact, the user attempted to render their negative content illegible to political censorship. This case starkly illustrates the severe impact of platform censorship on individuals’ depression and suicidal expression. Additional stress is caused by the police’s invasive monitoring of personal social media, intensifying the tension between the state’s interest in maintaining social harmony and individuals’ rights to express their mental struggles.

The visibility of Feiyang’s protest posts on social media plays a critical role in the negotiation of discourses surrounding depression. The comments on this incident can be categorized into three types. The first category consists of malicious insults, such as “You clearly have problems; people like you should be forcibly institutionalized in a psychiatric hospital.” The second category expresses sympathy, with comments like “I’ve faced similar issues, and it was incredibly frustrating. They do not solve the problem; they just want it to disappear” or “I once posted similar content under an anonymous account, and the police came to my door. Even my tutor, family, and classmates found out. It was so annoying.” The third category involves collective protests, with statements like “China’s socialism does not allow you to be sick,” and “If we cannot solve the problem, we solve the person who raised it; that’s a long-standing traditional virtue in China.”

The police’s framing of Feiyang’s posts as expressions of “pessimism,” together with their actions of notifying the individual’s school teachers and family members, further exemplifies the official discourse of individualism legitimacy. Although the discourse of harmonious screening emphasizes timely detection and assistance, its implementation by local police remained largely focused on regulating visibility of Weibo posts rather than providing substantive support. In this case, institutional intervention was limited to requesting the removal of suicidal posts and informing the individual’s families and teachers. Rather than addressing the structural and emotional dimensions of depression, such practices functioned to restore a “harmonious” public sphere by reducing the visibility of distressing expressions. This case illustrates the instrumental use of invisibility as a mode of governance and reveals institutional distrust toward individuals’ depressive agency of freely venting their fragile sentiments.

#### Vulnerability disclosure

Individuals’ narratives of their depression experiences often involve strong venting of negative emotions that may differ from their interactions with familiar people. The anonymous and public attributes of Weibo afford users the opportunity to post their depressive experiences and seek connection with others. Self-disclosure of depression on social media facilitates their storytelling and real emotions in a spontaneous and reflective manner. Many users feel that Weibo is more private. For instance, one Weibo user shared a personal confession:

*I’m posting my depression because I know no one on Weibo knows me and I can tell the truth. During the day I am an optimistic happy person in everyone’s eyes, but in fact, I cannot feel happy (…).*

This quote illustrates how the perceived anonymity of the platform enables users to express emotions that are suppressed in their offline lives, highlighting the complex interplay between visibility, identity, and emotional disclosure on social media. Many users mentioned the concept of “smiling depression,” describing a facade of happiness that conceals inner turmoil. These posts serve as a crucial outlet for emotional release, as well as a call for social understanding and support.

Depressive individuals reflect on the meaning of human vulnerability and fragile emotions, contributing to a deeper understanding of mental issues. These discussions allow for the fostering of a culture of empathy and proactive mental health care at the societal level. Many netizens have expressed condolences for Gaoli with communication about the deceased ordinary figure offering a way for individuals to disclose their own suicidal emotions (Yang et al., 2022). On the other hand, this dialogue also fosters a greater respect for death. For instance,

*(…) Being alone is really lonely, but I can only be alone. I need to live well, not self-harm, avoid any abnormal emotions, not cry or resist, and certainly not give up my studies. But I often feel like dying. (…)*

Having a social media space to express emotions and pain, addressing issues like vulnerability, allows users to resonate with each other, expressing their pain and seeking solace. Emotional communication can easily spread and generate resonance, forming both individual and collective memories.

Some users, after posting negative emotions, stop posting for long periods. In some cases, other users leave comments asking, “It’s been a while, are you okay?” or “Hug, I understand how you feel.” Such vulnerability disclosures highlight individuals’ depressive agency, manifesting their ability to control, communicate, and seek support when experiencing depression. However, this individual self-disclosure of vulnerability and the desire to freely vent fragile emotions sometimes stand in contrast to official discourses advocating positive treatment.

### Connected resonance and rationalization

#### Empathetic support

Against the stigmatization of depression, new discursive interactions not only help reduce the stigma but also promote societal understanding and acceptance of depression. Our analysis identified three types of meaning and value exchange on Weibo that contribute to the reduction of depression stigma in the process of socio-cultural reconfiguration.

First, users share personal experiences with the diagnosis and treatment of depression. This includes detailed accounts of medication regimens and misdiagnoses, physical symptoms such as insomnia and crying, and the emotional journey through depression. The visibility and dissemination of emotional communication enhance discussions about diagnostic processes, which not only help others determine whether they have depression but also encourage people to actively seek help. As a user posted on the Depression Super Topic, “Insomnia is a significant symptom of both depression and anxiety. Anxiety and depression can both cause sleep disturbances”.

Second, users offer advice and support to online friends struggling with depression. The exchange of personal narratives about depression on social media plays a critical role in raising awareness and fostering a sense of community among those affected. This process breaks down the discrimination and disapproval of depression, circulating discursive inclusion of mental illnesses. Increasingly, users urge others not to ignore depression, to see a doctor actively, seek help from family and friends. Here is an example from the Depression Super Topic,

*If you encounter difficulties and lose enthusiasm for life, please be sure to speak out or write it down. Let your family and friends know, go out with them, talk about your deepest thoughts, and find something you love to persist in. As you keep doing it, you’ll find the gloom lifting, and you’ll become more confident and cheerful (…).*

These findings are consistent with Li and Xu’s (2025a) study of community building among Chinese depression sufferers and Li and Xu’s (2023) analysis of coping strategies, including providing support, seeing a doctor, and seeking support from family, friends, and peers.

Third, users keep analyzing causes of depression struggles and discussing solutions. Unlike previous studies, which found that citizen journalists attribute depression more to workplace policies (Pan et al., 2018), the causes we found in the data include dysfunctional family dynamics, high sensitivity in personality, inability to integrate into groups, discrimination from groups, feeling unimportant, disliking one’s living circumstances, fear of new environments, concerns about burdening family, excessive time on social media without real-life interactions, marital issues, and postpartum depression or anxiety about child-rearing, etc. Factors such as family dynamics, personal sensitivity, social integration issues, and life transitions are frequently mentioned. These findings partly overlap with Xu and Li’s (2023) study, which identified parental control or neglect, school bullying, study or work stress, social norms, perfectionism, and limited self-disclosure as causes through which Chinese individuals living with depression made sense of their illness, while our data additionally highlight excessive social media use without real-life interactions, marital issues, and postpartum depression or anxiety about child-rearing.

The three types of interactive discussions identified in our dataset reflect users’ agency in managing depression, as well as their capacity to spontaneously show empathy, seek support, and challenge stigma. The visibility of these posts enhances the discourses of depression, transforming individual experiences into collective awareness.

#### Tactical rationalization

In the discursive practices of connected resonance and rationalization, users’ digital literacy to negotiate the visibility of their depression discussions reveals two primary strategies: challenge-posting and visibility-resetting. For challenge-posting, self-expression and the desire for visibility drive users to post on social media despite facing censorship by the state and platform when their content is deemed “overly negative” and not aligned with mainstream positive values. In response, users adopt two main strategies to challenge and resist invisibility. One is to post re-coding content in protest, as Feiyang did, posting screenshots of dialogues, using homophonic Chinese characters, and employing Chinese phonetic abbreviations.

Another way of challenge-posting involves retweeting pre-existing posts to their own accounts when faced with censorship closures. After comments on the Gaoli account were disabled by the platform, many users continued to retweet the post and share their emotional thoughts, though this diminished the reach of their distress signals. In general, comments left on popular Weibo posts have more visibility than retweets (Yin and Xie, 2024). These tactics reflect a form of digital resistance against state- and platform-imposed invisibility.

### Manifesting depressive agency

Drawing on the six discursive codes identified in our analysis, we develop the concept of “manifesting depressive agency” to capture how individuals and communities actively claim the need, right, and autonomy to make depressive experiences visible and participate in public mental-health discourse. Unlike an individualized understanding of agency as independent action, manifesting depressive agency emphasizes that agency is relationally produced through interactions among individuals, platform infrastructures, and institutional power. Under conditions of platform governance and censorship, individuals continuously negotiate how depressive experiences become publicly visible.

#### Claiming visibility under institutional regulation

The first dimension of manifesting depressive agency concerns individuals’ attempts to reclaim visibility against institutional frameworks that regulate depressive expressions. Our findings reveal a structural tension within official depression discourse. Through harmonious screening, state institutions promote “timely detection and intervention” as a form of public mental-health governance. However, depressive and suicidal expressions often challenge the dominant discourse of social harmony and “positive energy” by revealing individual suffering and social distress. Therefore, while screening practices are justified through governmental interventions, they may simultaneously marginalize individual depressive vulnerability.

This tension is further reflected in the discourse of individualism legitimacy. Consistent with previous research showing that Chinese state-owned media frequently frame mental-health issues through individual responsibility and self-management (Pan et al., 2018; Zhang et al., 2021), official narratives tend to position depression as an individual condition requiring personal adjustment and institutional guidance. Such framing legitimizes individual suffering only when it remains compatible with existing social norms, thereby limiting the extent to which depressive experiences can function as public claims.

The discourse of citizenship protest demonstrates how users challenge these limitations by demanding legitimacy for diverse representations of depression. Feiyang’s case, together with users’ comments of critical responses toward official intervention practices, illustrates how individuals resist excessive regulation of depressive expression. This finding resonates with Yan’s (2010) observation that political education may function as a corrective mechanism for mental illness. However, our study extends this argument by showing how such governance tensions are negotiated within digital spaces: users contest institutional definitions of appropriate depression expression and advocate for the right to publicly articulate distress. In this sense, manifesting depressive agency involves not only emotional disclosure but also a struggle over who has the authority to define legitimate depressive experiences.

#### Mobilizing platform affordances for depressive visibility

The second dimension of manifesting depressive agency concerns how users strategically employ platform affordances to make depression visible and construct supportive networks. Through vulnerability disclosure, individuals transform social media into a space for sharing personal suffering and seeking recognition from others. Previous studies have shown that online disclosure can reduce stigma by allowing individuals to connect with strangers who share similar depressive experiences (Hendry, 2020; Li et al., 2024; Li and Xu, 2025a; Wang and Liu, 2016). Our findings further demonstrate that such disclosure represents an active process of visibility production: users do not merely reveal vulnerability but actively seek relational recognition within platform environments.

The discourse of empathetic support further illustrates how individual disclosures develop into collective meaning-making practices. By framing depression as a common and legitimate health condition, users provide emotional affirmation and construct supportive communities around shared vulnerability. These interactions generate connected action through discursive resonance, increasing the visibility and legitimacy of depression discussions. This finding aligns with Li and Xu’s (2025a, p.1) argument that online communities construct “negative individual identities and positive collective identities” through shared participation and collective meaning-making.

The discourse of tactical rationalization reveals how users negotiate platform visibility rules to sustain depressive discourse. As algorithmic mechanisms, trending hashtags, and celebrity attention influence content visibility (Yin and Xie, 2024; Zhang et al., 2023), users strategically adapt their communication practices by engaging with highly visible topics and leveraging celebrity disclosures, such as Meiming’s posts. Through these practices, users frame depression as a legitimate illness rather than a personal weakness. This finding extends previous research on celebrity disclosures and mental-health communication by demonstrating that celebrity visibility does not simply influence public attitudes. It also provides users with strategic opportunities to negotiate their own visibility and legitimacy (Francis, 2018; Hoffner, 2020; Leung, 2019; Park and Hoffner, 2020).

#### Manifesting depressive agency as an ongoing negotiation

Together, these findings conceptualize manifesting depressive agency as a continuous negotiation among individual expression, platform visibility mechanisms, and institutional governance. Individuals with depression seek the right and autonomy to make their experiences visible, yet such visibility is not guaranteed. Instead, users navigate platform rules, algorithmic amplification, and institutional interventions through practices such as vulnerability disclosure, collective resonance, challenge-posting, and visibility-resetting. These tactics align with the concept of “movement visibility, resonance, and legitimacy” (Li et al., 2024, p. 2). The connected discursive interactions rationalize depression discussions, showcasing their resonance and offering support (Hendry, 2020; Li and Xu, 2025a).

Therefore, manifesting depressive agency does not represent unrestricted freedom of expression. Rather, it describes how marginalized health voices strategically negotiate visibility within constrained digital environments. By incorporating platform visibility into discourse analysis, our study demonstrates that mental-health discourse on social media emerges from the interaction between platform power and user agency. This perspective extends existing research on digital mental-health communication by shifting attention from whether social media empowers or constrains users toward understanding how users continuously construct, challenge, and reshape the conditions under which depressive experiences become publicly recognizable.

## Discussion

Social media affords depressive individuals to freely exchange meaningful experiences, seek emotional communication and support under human subjectivity, agency and values (Wang et al., 2023). This study offers a contribution to the understanding of complex dynamics shaping depression discourses on Chinese social media. We use platformized discourse analysis as a sampling and analytical framework that critically examines how platform visibility structures shape discursive formations. This approach requires researchers to navigate the platform visibility assemblage and implement stratified sampling and coding according to different levels of visibility, in order to capture diverse discourses. Sampling across account types enriches the dataset and facilitates cross-checking of discursive codes.

This study offers several theoretical implications. The concept of manifesting depressive agency highlights that mental-health communication is not merely shaped by institutional narratives or platform architectures but also by users’ continuous efforts to make vulnerable experiences publicly recognizable. By examining the interplay between top-down influences from state intervention and bottom-up initiatives by ordinary users, this research uncovers a nuanced and paradoxical landscape of depression discussions. Our findings reveal that both the state and individuals share a common theme in destigmatizing depression and providing support for individuals experiencing depression, but they diverge in the paths to achieve this goal, sometimes leading to conflicts. The state’s approach often frames mental health as a personal or familial failing rather than a societal issue (Pan et al., 2018; Yang et al., 2007; Zhang et al., 2021).

Beyond the existing literature that primarily focuses on the need for responsible online messaging (Wang and Liu, 2016), this study uncovers the inherent tension between state-driven screening and citizens’ needs of freely vent fragile sentiments. The vernacular discourse is reflected in individuals’ desire to express their depressive agency in sharing their vulnerable emotions and shaping public discourse, a process we summarize as “manifesting depressive agency.” This concept refers to the ongoing process through which individuals and communities claim, negotiate, and sustain the visibility and legitimacy of depressive experiences within platformized public discourse. It captures how users exercise agency not only through emotional disclosure but also through strategic engagement with platform affordances and negotiation with institutional visibility regimes.

Our findings highlight several implications for mental health governance and digital platform design. First, governmental authorities and social institutions should recognize depressive agency as an essential component of effective mental health communication. While monitoring and intervention mechanisms are necessary for suicide prevention and crisis response, overly restrictive approaches that prioritize visibility reduction may unintentionally discourage help-seeking behaviors or reinforce stigma. A more supportive approach should balance risk management with respect for individuals’ autonomy, emotional expression, and participation in public discourse.

Second, mental health interventions should move beyond detection-oriented models toward more collaborative and care-oriented approaches. Feiyang’s case illustrates that institutional responses relying primarily on moral persuasion and content removal may be insufficient when individuals express complex emotional distress online. Strengthening collaboration among schools, community organizations, social workers, and professional mental health services could enable more empathetic responses that address both immediate risks and underlying social pressures.

Third, digital platforms should take more responsibility in shaping healthier visibility environments. Rather than treating depression-related content solely as risky or harmful information requiring suppression, platforms could develop more nuanced governance mechanisms, such as providing accessible mental health resources, improving crisis-response pathways, and supporting constructive peer interactions. Such approaches would transform platform visibility from a mechanism of regulation into a resource for public mental health support.

Finally, grassroots online communities represent an important complementary resource in mental health communication. As demonstrated through empathetic support and connected resonance, user-generated interactions can reduce stigma and provide emotional recognition, particularly when professional resources are limited. However, these peer-based networks should be viewed as complementary rather than substitutes for professional care. Policies and platform designs should support responsible community engagement while ensuring pathways to formal mental health services.

## Limitations

This study has several limitations. First, platformized discourse analysis relies on researchers’ sustained engagement with the research topic, requiring long-term observation of both platform environments and individual voices that may become less visible due to platform mechanisms. In this study, conventional keyword-based sampling approaches failed to capture cases such as Feiyang and Gaoli because platform-induced invisibility constrained their retrievability. Therefore, although our visibility-oriented sampling strategy enables access to marginalized and less visible discourses, it may also introduce researcher interpretive bias and subjective judgments in identifying relevant cases.

Moreover, our findings are derived from a relatively limited number of cases and should not be interpreted as statistically representative of depression discussions on Weibo or other social media platforms. Instead, this study aims to provide an in-depth interpretation of how depression discourses are constructed, circulated, and negotiated within specific platform contexts. Future studies could complement social media data with additional sources, such as interviews with users, mental health professionals, platform moderators, and institutional officers. Such data triangulation and methodological triangulation would provide a more comprehensive understanding of the complex relationship between psychological health communication, platform governance, and user agency.

Second, this study has several potential sources of bias, including algorithmic bias, sampling bias, and limitations concerning the representativeness of social media users. Because platform algorithms determine which content becomes visible and retrievable, social media data cannot fully represent the experiences of individuals whose voices remain marginalized or excluded from digital spaces.

The sampling period was limited to January to August 2023. Future research could extend the temporal scope to examine how depression discourse evolves across different social and policy contexts. In addition, future studies could adopt more stratified sampling strategies by incorporating diverse institutional and individual actors, such as the National Health Commission, mental health practitioners, online mental health influencers, and other depression-related public events. Expanding the range of actors would further illuminate how different visibility regimes shape mental health communication.

## Data Availability

The raw data supporting the conclusions of this article will be made available upon request.
